# Myocardial Injury in Aneurysmal Subarachnoid Haemorrhage: Clinical Correlates and Impact on Mortality in a Single-Centre Australian Intensive Care Unit Cohort

**DOI:** 10.7759/cureus.100820

**Published:** 2026-01-05

**Authors:** Abdelghafar Sharara, Shyamala Sriram, Hesham Abdelwahed

**Affiliations:** 1 Intensive Care Unit, The Royal Melbourne Hospital, Melbourne, AUS; 2 Critical Care, Maitland Hospital, Newcastle, AUS; 3 Critical Care Medicine, Newcastle University, Newcastle, AUS; 4 Critical Care, Wollongong Hospital, Wollongong, AUS

**Keywords:** acute myocardial injury, aneurysmal subarachnoid haemorrhage, cerebral vasospasm, delayed cerebral ischemia (dci), intensive care unit

## Abstract

Background

Myocardial injury is a recognised complication of aneurysmal subarachnoid haemorrhage (aSAH), arising from catecholamine-mediated neuro-cardiac mechanisms. While international data describe variable incidence and clinical significance, contemporary Australian evidence remains limited. This study aimed to determine the incidence, predictors, and clinical impact of myocardial injury following aSAH in a tertiary Australian intensive care unit (ICU).

Methods

We performed a retrospective cohort study of adults admitted with aSAH to the Royal Melbourne Hospital ICU between 1 June 2020 and 31 May 2025. Myocardial injury was defined by multimodal criteria: elevated high-sensitivity troponin, new ECG abnormalities, or echocardiographic dysfunction within 72 hours. Outcomes included ICU and hospital mortality, length of stay (LOS), and neurovascular complications.

Results

Among 300 patients (mean age 56.9 years; 66% female), myocardial injury occurred in 179 (59.7%). Transthoracic echocardiography was performed in 121 patients (40.3%), of whom 74 (24.7%) demonstrated abnormal findings. Elevated troponin was observed in 31.7% (n=95), prolonged QTc in 29% (n=87), and new-onset arrhythmia in 16.7% (n=50). Patients with myocardial injury presented with more severe neurological impairment [mean World Federation of Neurological Societies (WFNS) grade 4 vs. 2; GCS 12 vs. 14] and had higher rates of arrhythmia (36 patients, 20.1% vs. 14 patients, 11.6%), symptomatic vasospasm (73 patients, 40.8% vs. 29 patients, 24.0%), and hydrocephalus (124 patients, 69.3% vs. 64 patients, 52.9%). ICU mortality was significantly higher in the myocardial injury group (44 patients, 24.6% vs. 14 patients, 11.6%; p=0.008), as was hospital mortality (60 patients, 33.5% vs. 16 patients, 13.2%; p=0.0001). Logistic regression identified symptomatic vasospasm as an independent predictor of myocardial injury [odds ratio (OR) 5.1, p<0.001; model accuracy 78.6%, area under the curve (AUC) 0.771].

Conclusions

Myocardial injury was frequent in this Australian aSAH cohort and correlated with greater neurological severity, neurovascular complications, and higher mortality.

## Introduction

Aneurysmal subarachnoid haemorrhage (aSAH) remains a life-threatening neurovascular event characterised by high early mortality and significant morbidity from both brain injury and systemic complications [1]. Epidemiological data from Australia and New Zealand show an incidence of approximately nine to 10 per 100 000 person-years and a case fatality of around 30% [2].

Beyond the primary neurological insult, one of the most significant systemic sequelae is cardiac dysfunction, which may present as myocardial injury, arrhythmia, QTc prolongation, or transient ventricular systolic impairment [3,4]. These abnormalities are thought to result from catecholamine-mediated myocardial toxicity and sympathetic overactivation, producing stress (Takotsubo-type) cardiomyopathy [5].

Previous studies have demonstrated variable incidence and prognostic impact. Meta-analytic data suggest that around 20% of patients develop echocardiographically confirmed cardiac dysfunction, which independently predicts higher in-hospital mortality [1]. Large international cohorts have also linked troponin elevation and wall-motion abnormalities to delayed cerebral ischemia (DCI) and poor outcomes [6]. However, findings remain inconsistent, and the true burden and predictors of myocardial injury likely differ across populations and monitoring practices.

Prior studies have variably defined myocardial injury using cardiac biomarkers alone, echocardiographic dysfunction, or a combination of modalities; accordingly, we adopted a pragmatic multimodal definition to capture the broader spectrum of neurogenic cardiac manifestations described after aSAH.

Despite well-established neurocritical care networks, contemporary Australian data on the incidence, risk factors, and clinical implications of myocardial injury after aSAH are scarce. Existing registries have largely focused on neurological outcomes, with limited characterization of systemic complications [7,8].

The primary objective of this study was to quantify the incidence of myocardial injury in patients with aneurysmal subarachnoid haemorrhage admitted to a tertiary Australian intensive care unit (ICU). Secondary objectives were to identify clinical predictors of myocardial injury and to examine its adjusted association with in-hospital outcomes, including mortality and hospital resource utilisation.

## Materials and methods

Study design and setting

This single-centre retrospective cohort study was conducted in the ICU at The Royal Melbourne Hospital, a tertiary referral centre in Australia. Following ethical approval (QA2025076), all patients admitted to the ICU between June 1, 2020, and May 31, 2025, with a diagnosis of aSAH were included.

Participants

Adult patients aged 18 years and older admitted to the ICU at The Royal Melbourne Hospital during the study period were eligible for inclusion if they had a confirmed diagnosis of aSAH. The diagnosis was established using computed tomography (CT) brain imaging with CT angiography and/or digital subtraction angiography (DSA). Eligible patients were those admitted to the ICU during their index hospitalisation for the management of aSAH, either before or after aneurysm securing. Patients were excluded if they had traumatic subarachnoid haemorrhage, SAH secondary to a structural lesion without a ruptured aneurysm (such as an isolated arteriovenous malformation or dural arteriovenous fistula), or pure perimesencephalic non-aneurysmal SAH. Readmissions during the same hospitalisation were not considered, and only the initial ICU admission was included in the analysis.

Data collection and variables

Data were extracted from the hospital’s electronic medical records, the ICU clinical information system, and radiology and cardiology databases. For each patient, baseline demographic and clinical characteristics were collected, including age, sex, comorbidities, and any history of pre-existing cardiac disease. Neurological and neurosurgical data encompassed the World Federation of Neurological Societies (WFNS) grade [9], modified Fisher grade [10], aneurysm site and size, securing modality (clipping, coiling, or combined), and complications related to aSAH, such as symptomatic vasospasm, rebleeding, seizures, hydrocephalus, DCI, and ventriculitis.

ICU-related parameters included the Acute Physiology and Chronic Health Evaluation II (APACHE II) score on admission, requirement and duration of mechanical ventilation, use of vasopressors or inotropes, and ICU and hospital length of stay. Mortality was recorded at both ICU and hospital discharge. Cardiac investigations encompassed high-sensitivity cardiac troponin (hs-cTn), B-type natriuretic peptide (BNP), electrocardiography (ECG), and transthoracic echocardiography (TTE). These data were collected for the first seven days following ictus, with a prespecified primary assessment window of 72 hours from ICU admission, corresponding to the typical time course of neurogenic cardiac injury after aSAH.

Data extraction was performed retrospectively by investigators with routine clinical access to ICU, pathology, cardiology, and radiology systems using a standardised data collection template. Objective data fields, including laboratory values, ECG reports, echocardiographic measurements, and clinical outcomes, were obtained directly from source records. Given the retrospective nature of the study, formal blinding and inter-rater reliability assessment were not performed.

Cardiac assessments

Cardiac evaluation during the first 72 hours of ICU admission included biomarker analysis, electrocardiography, and transthoracic echocardiography. High-sensitivity cardiac troponin was measured on ICU admission and subsequently at the discretion of the treating clinicians. Each patient underwent at least one 12-lead ECG within 24 hours of ICU admission, with additional recordings performed for clinical indications such as haemodynamic instability, chest pain, arrhythmia, or rising cardiac biomarkers. Transthoracic echocardiography was conducted at the bedside by cardiology services or accredited intensivists, typically within 72 hours of ICU admission, and repeated in cases of haemodynamic deterioration, new-onset arrhythmia, or suspected cardiogenic shock.

Definition of myocardial injury

Myocardial injury was defined as the presence of at least one of three diagnostic categories-biochemical, electrocardiographic, or echocardiographic-occurring within the first 72 hours of ICU admission [1,11,12]. This pragmatic definition was chosen to reflect the spectrum of neuro-cardiac manifestations described in aneurysmal subarachnoid haemorrhage, acknowledging that some prior studies have defined myocardial injury using cardiac biomarkers alone.

Biochemical myocardial injury was defined by at least one hs-cTn value exceeding the 99th percentile upper reference limit (URL) for the local assay. The manufacturer-reported 99th percentile URLs were 14 ng/L for high-sensitivity cardiac troponin T [Roche Elecsys (Roche Diagnostics International Ltd., Rotkreuz, Switzerland)] and approximately 26 ng/L for men and 16 ng/L for women for high-sensitivity cardiac troponin I [Abbott Architect/Alinity (Abbott Diagnostics, Abbott Park, United States). The precise cut-offs applied in this study followed the reference values used by the Royal Melbourne Hospital pathology service.

Electrocardiographic evidence of myocardial involvement was identified by any new abnormality on a 12-lead ECG not attributable to pre-existing cardiac disease. Abnormalities included ST-segment elevation or depression of at least 1 mm in two or more contiguous leads, new T-wave inversion in two or more contiguous leads, the presence of new pathological Q waves, QTc prolongation defined as a QTc interval of at least 500 ms or an increase of 60 ms from baseline, or new clinically significant arrhythmia such as atrial fibrillation or flutter, supraventricular tachycardia, ventricular tachycardia or fibrillation, or high-grade atrioventricular block.

Echocardiographic evidence of stress-related cardiac dysfunction was defined by any new abnormality on TTE not explained by known pre-existing cardiac disease. This included left ventricular systolic dysfunction, defined as a left ventricular ejection fraction (LVEF) below 50% or an absolute reduction in LVEF of at least 10 percentage points compared with any prior pre-ictus echocardiogram, when available. Regional wall-motion abnormalities in a non-coronary distribution consistent with neurogenic or Takotsubo-like cardiomyopathy were also considered diagnostic. A Takotsubo pattern was characterised by typical or atypical ballooning (apical, mid-ventricular, or basal) with hypercontractile segments elsewhere, in the absence of a culprit coronary occlusion when angiography was performed. Right ventricular systolic dysfunction was considered evidence of myocardial injury if deemed related to acute aSAH rather than chronic pulmonary vascular or right-sided cardiac disease.

Outcomes

The primary outcome of this study was the incidence of myocardial injury within the first 72 hours of ICU admission, defined according to the criteria described above. Secondary outcomes included an evaluation of the clinical characteristics and course of patients with aneurysmal subarachnoid haemorrhage managed at our centre, as well as the association between myocardial injury and key clinical outcomes, specifically ICU length of stay, hospital length of stay, and mortality at both ICU and hospital discharge.

Statistical analysis

Statistical analyses were performed using Python version 3.X [Python Software Foundation (PSF), Wilmington, Delaware, United States] with pandas (open-source Python library maintained by the pandas development community), SciPy (open-source Python library maintained by the SciPy community), statsmodels (open-source Python library maintained by the statsmodels development community), and scikit-learn (open-source Python library maintained by the scikit-learn development community) libraries. Continuous variables were assessed for normality using the Shapiro-Wilk test and presented as mean ± SD or median [interquartile range (IQR)], while categorical variables were expressed as frequencies and percentages. Group comparisons were conducted using the Student’s t-test or Mann-Whitney U test for continuous variables and the Chi-square or Fisher’s exact test for categorical variables. Correlations were assessed with Spearman’s rank coefficient, and variables with p<0.1 on univariable analysis were entered into a binary logistic regression model to identify independent predictors of myocardial injury. Statistical significance was set at a two-tailed p<0.05.

## Results

Study population and baseline characteristics

Following ethical approval, we conducted a retrospective analysis of the electronic medical records of all patients admitted to the Royal Melbourne Hospital ICU with aSAH between May 1, 2020, and May 31, 2025. The study included 300 patients.

The cohort had a mean age of 56.9 years (SD±14.8), with two-thirds (66%, n=198) being female. The mean Glasgow Coma Scale (GCS) score on admission was 10.9 (SD±4.3), and the mean World Federation of Neurosurgical Societies (WFNS) grade was 3.0 (SD±1.6). The mean aneurysm size was 6.8 mm (SD±4.4). The most common comorbidity was hypertension (36%, n=108). Detailed baseline characteristics, complications, and outcomes are presented in Table 1.

**Table 1:** Baseline Characteristics and Clinical Outcomes of the Study Cohort. MV: mechanical ventilation, ICU: intensive care unit, LOS: length of stay, SD: standard deviation, MM: millimetre, mS: millisecond. Y: yes, N: nO, NA: not available, MV: mechanical ventilation, GCS: Glasgow Coma Scale, WFNS: World Federation of Neurosurgical Societies.

	Numbers (percentages)	Mean ± SD
Age (years)		56.98 ±14.80
Gender		
- Male	102/300 (34%)	
- Female	198/300 (66%)	
Comorbidities		
- Hypertension	108/300 (36%)	
- Diabetes	35/300 (11.6%)	
- Smoking	20/300 (6.7%)	
- Stroke/TIA	22/300 (7.3%)	
GCS on admission		10.90 ± 4.3
WFNS on admission		3.03 ± 1.6
Fisher score		3.70 ± 0.7
Aneurysm size (MM)		6.8 ± 4.4
QTC (ms)		506.14 ± 33.9
Troponin Elevated	95/300 (31.7%)	
Troponin (Data not available)	135/300 (45%)	
Troponin Not Elevated	70/300 (23,3%)	
QTc prolongation Present	87/300 (29%)	
QTc prolongation N	166/300 (55.3%)	
QTc prolongation (data not available)	47/300 (15.7)	
Arrhythmia Present	50/300 (16.7%)	
Arrhythmia N	203/300 (67.7%)	
Arrhythmia (Data not available)	47/300 (15.7%)	
Symptomatic vasospasm Present	198/300 (66%)	
Symptomatic vasospasm N	102/300 (34%)	
Hydrocephalus Present	188/300 (62.7%)	
Hydrocephalus N	112/300 (37.3%)	
Delayed cerebral ischaemia Present	81/300 (27%)	
Delayed cerebral ischaemia N	219/300 (73%)	
Seizures Present	76/300 (25.3%)	
Seizures N	224/300 (74.7%)	
Duration of MV (days)		4.58 ± 4
ICU LOS (days)		5.99 ± 5.43
Hospital LOS (days)		20.96 ± 20.82
ICU mortality	58/300 (19.3%)	
Hospital mortality	76/300 (25.3%)	
Discharged home	74/300 (24.66%)	
Discharge to Rehab	115/300 (38.33%)	
Transferred to another hospital	32/300 (10.6%)	

Aneurysm location distribution

**Figure 1:**
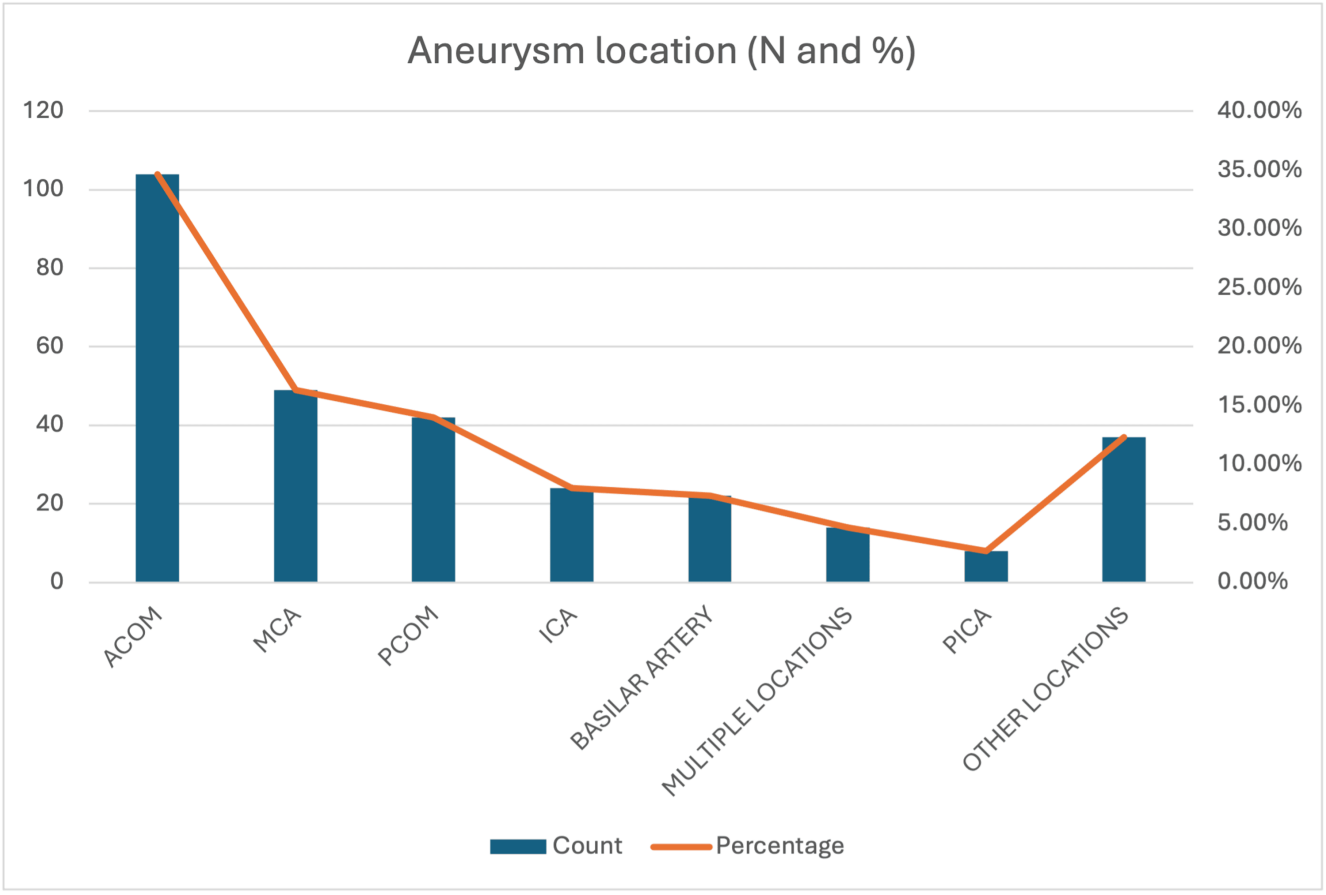
Bar Chart Distribution of Aneurysm Locations (N=300). Distribution of aneurysm locations in the study cohort (N=300). ACOM: anterior communicating artery, MCA: middle cerebral artery, PCOM: posterior communicating artery, ICA: internal carotid artery, BA: basilar artery.

The distribution of aneurysm locations within the cohort of 300 patients is detailed in Figure 1. The most frequent site was the anterior communicating artery (ACOM), accounting for 104 cases (34.7%). This was followed by aneurysms of the middle cerebral artery (MCA) in 49 patients (16.3%) and the posterior communicating artery (PCOM) in 42 patients (14.0%).

Clinical outcomes and complications

Symptomatic vasospasm occurred in 34% (n=102) of patients, hydrocephalus in 37.3% (n=112), and delayed cerebral ischemia (DCI) in 27% (n=81). The overall incidence of myocardial injury (MI) was 59.7%. Electrocardiographic and biomarker abnormalities were frequent, with elevated troponin observed in 31.7% (n=95), prolonged QTc in 29% (n=87), and new-onset arrhythmia in 16.7% (n=50). Transthoracic echocardiography was performed in 121 of 300 patients (40.3%), with regional wall motion abnormalities (RWMA), right ventricular dysfunction, or a Takotsubo-like pattern identified in 74 cases (24.7%). The ICU mortality rate was 19.3% (n=58), and in-hospital mortality was 25.3% (n=76).

Comparison between patients with and without myocardial injury

Patients were stratified into two groups: those with myocardial injury (Group 1, n=179) and those without (Group 2, n=121). Comparative analysis is summarized in Table 2. Arrhythmia, symptomatic vasospasm, and hydrocephalus were more common in the MI group, but seizures and DCI were not significantly different between the groups. Both ICU and hospital mortality were significantly higher in the MI group.

**Table 2:** Comparison of Characteristics and Outcomes by Myocardial Injury Status. MV: mechanical ventilation, ICU: Intensive Care Unit, LOS: length of stay, MM: millimetre, DCI: delayed cerebral ischaemia, GCS: Glasgow Coma Scale, WFNS: World Federation of Neurosurgical Societies. Continuous variables are presented as median (interquartile range) and were compared using the Mann–Whitney U test. Categorical variables are presented as number (percentage) and were compared using the Chi-square test. The test statistic column reports U values and χ² values, as appropriate.

Variable	Type	Category	Group with MI	Group without MI	Statistical test	Test statistic	p-value
Age (years)	Continuous		59.50 (48.60–68.78)	57.95 (43.40–67.10)	Mann–Whitney U test	U=11672.50	0.253
GCS on admission	Continuous		12.00 (6.00–14.00)	14.00 (10.50–15.00)	Mann–Whitney U test	U=5420.00	0.0005
WFNS grade	Continuous		4.00 (2.00–5.00)	2.00 (1.00–4.00)	Mann–Whitney U test	U=7378.50	0.0083
Aneurysm size (MM)	Continuous		6.00 (4.00–9.00)	5.50 (3.60–8.00)	Mann–Whitney U test	U=7036.00	0.2756
DurationofMV (days)	Continuous		3.00 (2.00–7.00)	2.50 (2.00–7.25)	Mann–Whitney U test	U=3563.50	0.9348
ICU length of stay (days)	Continuous		4.20 (2.30–8.15)	4.30 (2.60–9.00)	Mann–Whitney U test	U=10496.50	0.6519
Hospital length of stay (days)	Continuous		14.60 (6.45–24.00)	19.70 (12.80–26.20)	Mann–Whitney U test	U=8599.00	0.0025
Sex	Categorical	F	116 (64.8%)	82 (67.8%)	Chi-square test	χ²=0.17	0.6837
Sex	Categorical	M	63 (35.2%)	39 (32.2%)			
Troponin high (Y/N)	Categorical	N	35 (26.9%)	35 (100.0%)	Chi-square test	χ²=57.33	0
Troponin high (Y/N)	Categorical	Y	95 (73.1%)	0 (0.0%)			
QTc prolongation (Y/N)	Categorical	N	122 (70.1%)	44 (55.7%)	Chi-square test	χ²=4.39	0.0362
QTc prolongation (Y/N)	Categorical	Y	52 (29.9%)	35 (44.3%)			
Arrhythmias (Y/N)	Categorical	N	138 (79.3%)	65 (82.3%)	Chi-square test	χ²=0.14	0.7046
Arrhythmias (Y/N)	Categorical	Y	36 (20.7%)	14 (17.7%)			
Symptomatic vasospasm Y/N	Categorical	N	106 (59.2%)	92 (76.0%)	Chi-square test	χ²=8.36	0.0038
Symptomatic vasospasm Y/N	Categorical	Y	73 (40.8%)	29 (24.0%)			
Hydrocephalus Y/N	Categorical	N	55 (30.7%)	57 (47.1%)	Chi-square test	χ²=7.60	0.0059
Hydrocephalus Y/N	Categorical	Y	124 (69.3%)	64 (52.9%)			
Delayed cerebral ischaemia (DCI) Y/N	Categorical	N	139 (77.7%)	80 (66.1%)	Chi-square test	χ²=4.31	0.0379
Delayed cerebral ischaemia (DCI) Y/N	Categorical	Y	40 (22.3%)	41 (33.9%)			
Seizures Y/N	Categorical	N	130 (72.6%)	94 (77.7%)	Chi-square test	χ²=0.73	0.3935
Seizures Y/N	Categorical	Y	49 (27.4%)	27 (22.3%)			
Mortality – ICU	Categorical	N	135 (75.4%)	107 (88.4%)	Chi-square test	χ²=7.02	0.008
Mortality – ICU	Categorical	Y	44 (24.6%)	14 (11.6%)			
Mortality – Hospital	Categorical	N	119 (66.5%)	105 (86.8%)	Chi-square test	χ²=14.67	0.0001
Mortality – Hospital	Categorical	Y	60 (33.5%)	16 (13.2%)			

Patients with MI presented with a significantly worse neurological status, evidenced by a lower mean GCS (12 vs. 14, p=0.0005) and a higher mean WFNS grade (4 vs. 2, p=0.0083) on admission, reflecting an association with greater overall disease severity. The incidence of arrhythmia (20.1% vs. 11.6%, p<0.0001), symptomatic vasospasm (40.8% vs. 24%, p=0.0038), and hydrocephalus (69.3% vs. 52.9%, p=0.0059) were all significantly higher in the MI group. While the mean hospital length of stay was shorter for the MI group (14.6 vs. 19.7 days, p=0.0025), both ICU mortality (24.6% vs. 11.6%, p=0.008) and hospital mortality (33.5% vs. 13.2%, p=0.0001) were significantly higher.

Risk factor analysis for myocardial injury

Univariable Spearman’s rank correlation analysis revealed that a higher WFNS grade was modestly but significantly correlated with myocardial injury (ρ=0.17, p=0.008). Aneurysm location was also strongly associated with myocardial injury (p<0.00001), whereas age, sex, and aneurysm size showed no significant correlation. These associations were explored using a multivariable logistic regression model excluding aneurysm location, as shown in Table 3.

**Table 3:** Logistic Regression Analysis Before Adding Aneurysm Location Feature as Predictor. Multivariable logistic regression analysis of factors associated with myocardial injury. Regression coefficients, odds ratios (ORs), and 95% confidence intervals (CIs) were estimated using multivariable binary logistic regression with maximum likelihood estimation. Statistical significance was assessed using Wald z-tests. GCS: Glasgow Coma Scale, WFNS: World Federation of Neurosurgical Societies. Y/N_bin indicates binary-coded variables used for regression analysis (Yes=1, No=0).

Variable	Coefficient	Std. Error	z-value	p-value	OR	CI Lower (95%)	CI Upper (95%)
const	2.198152765	1.196609576	1.836984101	0.066212243	9.008357568	0.863156805	94.01594889
GCS on admission	-0.134271927	0.06695382	-2.005440871	0.044915936	0.874352272	0.766823207	0.99695978
WFNS grade	0.046782897	0.166551498	0.280891479	0.778793637	1.047894483	0.756046769	1.452400688
Hospital length of stay (days)	0.0028688	0.010249951	0.279884291	0.779566279	1.002872919	0.982926695	1.023223906
Arrhythmias (Y/N)_bin	0.395130831	0.419161416	0.942669853	0.345849798	1.484578407	0.652848326	3.375934283
Symptomatic vasospasm Y/N_bin	1.627047536	0.456189704	3.566602936	0.000361639	5.088827935	2.081173978	12.4430586
Hydrocephalus Y/N_bin	-0.231250765	0.376672562	-0.613930474	0.539261271	0.793540449	0.379266779	1.660325871
Delayed cerebral ischaemia (DCI) Y/N_bin	-1.913417805	0.454715309	-4.207946745	2.57702E-05	0.147575141	0.06052835	0.359805319

**Figure 2:**
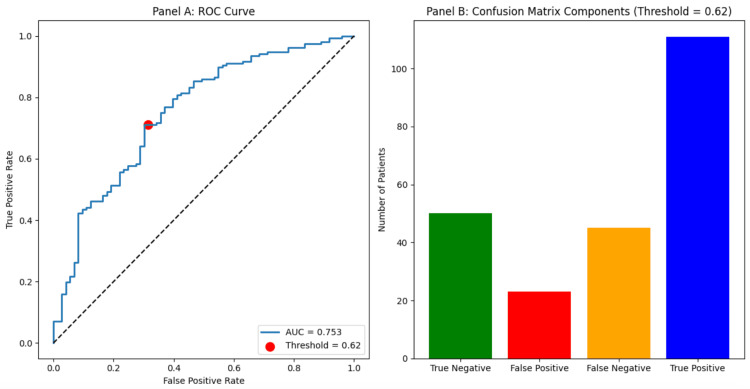
Discriminative Performance of the Multivariable Logistic Regression Model for Predicting Myocardial Injury Prior to Inclusion of Aneurysm Location as a Predictor. (A) Receiver operating characteristic (ROC) curve demonstrating the ability of the multivariable model to discriminate between patients with and without myocardial injury. The model includes Glasgow Coma Scale (GCS) on admission, World Federation of Neurosurgical Societies (WFNS) grade, hospital length of stay, arrhythmias, symptomatic vasospasm, hydrocephalus, and delayed cerebral ischaemia. The area under the curve (AUC) reflects overall model discrimination. (B) Confusion matrix showing the distribution of true negatives, false positives, false negatives, and true positives at the selected probability threshold of 0.62, illustrating the balance between sensitivity and specificity used for classification.

To identify independent predictors, we performed a logistic regression analysis. The initial model identified symptomatic vasospasm (OR≈5.1, p<0.001) as a strong predictor of MI, while DCI was associated with a reduced odds of MI (OR≈0.15, p<0.001). A lower GCS on admission was of borderline significance (OR≈0.87 per point, p=0.045). This model had an accuracy of 70.3% and an area under the receiver operating characteristic curve (AUC) of 0.753 (Figure 2 and Table 4).

**Table 4:** Multivariable Logistic Regression Analysis After Inclusion of Aneurysm Location (arterial site) as a Predictor of Myocardial Injury. Multivariable logistic regression analysis of factors associated with myocardial injury. Regression coefficients, odds ratios (ORs), and 95% confidence intervals (CIs) were estimated using multivariable binary logistic regression with maximum likelihood estimation. Statistical significance was assessed using Wald z-tests. GCS: Glasgow Coma Scale, WFNS: World Federation of Neurosurgical Societies. Y/N_bin indicates binary-coded variables used for regression analysis (Yes=1, No=0).

Variable	Coefficient	Std. Error	z-value	p-value	OR	CI Lower (95%)	CI Upper (95%)
const	0.985638429	1.540226362	0.639930892	0.522217529	2.679522024	0.130922495	54.84037144
Age (years)	0.011779839	0.012429127	0.947760769	0.343251257	1.011849494	0.987498006	1.036801485
Sex_bin	0.021959727	0.367718195	0.059718903	0.952379516	1.022202617	0.497204091	2.101547853
GCS on admission	-0.130489107	0.070531946	-1.850070971	0.064303321	0.877666054	0.764350228	1.007781085
WFNS grade	0.104078061	0.174668758	0.595859625	0.551269008	1.109687075	0.78799273	1.562711629
Hospital length of stay (days)	0.005052191	0.010465478	0.482748228	0.629274515	1.005064975	0.98465912	1.025893716
Arrhythmias (Y/N)_bin	0.342921931	0.43743464	0.783938672	0.433076097	1.409058754	0.597838751	3.321040279
Symptomatic vasospasm Y/N_bin	1.652179274	0.46574169	3.547415464	0.000389031	5.218339638	2.094557545	13.00086916
Hydrocephalus Y/N_bin	-0.242256215	0.392894431	-0.616593659	0.537502778	0.78485506	0.363376734	1.695203375
Delayed cerebral ischaemia (DCI) Y/N_bin	-2.088628191	0.488079313	-4.279280305	1.87499E-05	0.123856927	0.047584602	0.32238451
ACOM	0.668821983	0.787311267	0.849501349	0.39560238	1.951936552	0.417162375	9.133269271
BASILAR ARTERY	-0.630700156	0.924788443	-0.681993986	0.495242754	0.532219034	0.086877848	3.260406512
ICA	0.506582579	0.900820516	0.562356841	0.573872918	1.659609908	0.283939757	9.700314882
MCA	0.56141182	0.863441085	0.65020281	0.515561226	1.75314588	0.322742138	9.523145938
OTHER	-0.013535821	0.900332204	-0.015034251	0.988004855	0.986555377	0.168949654	5.760837558
PCOM	0.094372999	0.831914903	0.113440688	0.909681183	1.098969584	0.215208006	5.611938744

**Figure 3:**
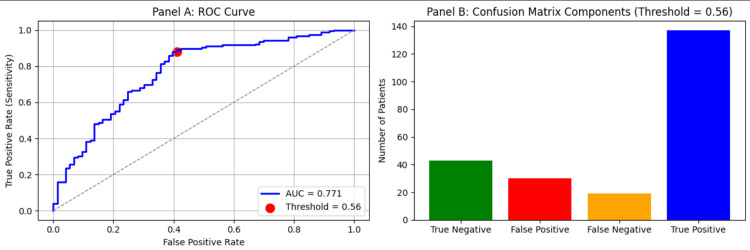
ROC curve for Multivariate Logistic regression after adding aneurysm location as a predictor to the model. (A) ROC curve for the multivariable logistic regression model predicting Myocardial Injury. Predictors: Glasgow Coma Scale (GCS) on admission, World Federation of Neurosurgical Societies (WFNS) grade, hospital length of stay, arrhythmias, symptomatic vasospasm, hydrocephalus, delayed cerebral ischaemia, and aneurysm location. The area under the curve (AUC) indicates model discriminative ability. (B) The confusion matrix is represented as counts of true negatives, false positives, false negatives, and true positive at the chosen threshold of 0.56.

In a subsequent model incorporating aneurysm location, overall predictive performance improved (Accuracy 78.6%, AUC 0.771). Within this model, symptomatic vasospasm and DCI remained strongly significant predictors (p<0.001 for both), while GCS became borderline non-significant (p=0.064). No individual aneurysm location emerged as a statistically significant independent predictor (Table 4 and Figure 3).

Impact of myocardial injury on resource utilisation and mortality

Myocardial injury (MI) was significantly associated with increased mortality. Patients with MI had higher ICU mortality (p=0.008) and greater in-hospital mortality (p=0.0001) compared with those without MI. Interestingly, MI was also correlated with a modestly shorter total hospital stay (Spearman’s r=-0.175, p=0.0023). In contrast, MI showed no significant association with ICU length of stay (r=-0.026, p=0.652) or duration of mechanical ventilation (r=0.006, p=0.934).

## Discussion

In this cohort of 300 patients with aSAH, two-thirds were female (66%), consistent with both Australian (62%) and international data (≈68%) [2,13,14]. The ACOM was the most common aneurysm location, accounting for one-third of the cases (104, 34.7%). The mean aneurysm size was 6.8 mm, which aligns with findings from similar cohorts [15,16].

The incidence of myocardial injury was 59.7% based on a multimodal definition incorporating troponin elevation, QTc prolongation, new arrhythmias, and echocardiography criteria. The rate is substantially higher than the previously reported in Australian cohorts, where the incidence is approximately 20% [17]. International data also show variable rates depending on the diagnostic criteria used. For example, Yousef et al. reported a 28% incidence [18], while a systematic review by Messina et al. found that one in five patients with aSAH developed cardiac dysfunction based on echocardiography criteria [1]. De Courson et al. have reported a 60% incidence of impaired LV systolic function using strain imaging [19]. and Mayer et al. [20], similarly reported echocardiographic evidence of myocardial dysfunction in 60% of their cohort. Urbaniak et al. observed myocardial injury in 50% of patients post-aSAH, defined by ECG abnormalities, elevated troponin, or abnormal echocardiography [21]. 

ECG abnormalities and troponin elevation were common, occurring in 45.6% and 31.7% of patients, respectively, figures comparable to prior international cohorts [17,22,23]. Patients with myocardial injury had higher WFNS grades and lower GCS scores, consistent with studies linking troponin elevation to poorer neurological condition and heightened catecholaminergic activation [18]. The association between myocardial injury and greater neurological severity supports the concept of neurogenic cardiac dysfunction as a marker of global physiological stress. Importantly, given the observational nature of this study, myocardial injury should be interpreted primarily as a marker of neurological and systemic disease severity rather than a direct causal determinant of adverse outcomes.

MI was strongly associated with increased mortality, with ICU and hospital mortality rates more than doubling among affected patients (24.6% vs. 11.6% and 33.5% vs. 13.2%, respectively). These findings are consistent with published literature demonstrating an increased risk of death among patients with troponin elevation or left ventricular dysfunction following aSAH. Ahmadian et al. [24] reported an even greater effect, noting a tenfold increase in mortality among patients with myocardial injury. Similarly, a meta-analysis by Van der Bilt et al. [25] found that myocardial injury was associated with increased risk of death, DCI, and poor neurological outcomes. Several other studies have supported these associations [6,23,26-28]. However, not all reports are concordant: for example, Urbaniak et al. [21] did not observe an increased mortality in the myocardial injury group, and this was echoed by Schulling et al. [29].

Interestingly, myocardial injury was also associated with higher rates of symptomatic vasospasm (40.8% vs. 24%) and hydrocephalus (69.3% vs. 52.9%), highlighting the link between severe neurovascular injury and systemic cardiac involvement. In contrast, delayed cerebral ischemia (DCI) was not more frequent in the myocardial injury group, which differs from several international datasets. This discrepancy may reflect survivor bias, under-recognition of DCI in the sickest patients, or variations in documentation and diagnostic triggers, rather than a true protective association. Notably, Van der Bilt et al. reported an increased risk of DCI following myocardial injury in a large meta-analysis. [25]

Overall cohort outcomes were consistent with contemporary high-income neurocritical care settings. ICU mortality was 19.3% and hospital mortality 25.3%, aligning with the expected 20-30% case-fatality rates reported in Australian, New Zealand, and European epidemiology study [2], and European epidemiology studies, as well as international cohorts [30-32]. Complication rates-including symptomatic vasospasm (34%), hydrocephalus (37%), seizures (25%), and DCI (27%)-were within published ranges [16,33,34].

In our multivariate regression analysis, symptomatic vasospasm emerged as a significant risk factor for MI, whereas all other variables were non-significant. This contrasts with the findings of Malik et al., who reported that older age, absence of hypertension, and smoking were independent predictors of MI [35].

Limitations

This study has several limitations. It was retrospective, single-centre, and non-randomised, which limits generalisability and precludes causal inference. Cardiac investigations were performed based on clinical indication rather than systematic screening; as a result, troponin testing and echocardiography were not available in all patients (approximately 45% and 60%, respectively). This may have introduced selection or surveillance bias, with cardiac abnormalities more likely to be identified in patients with greater illness severity, while subclinical myocardial injury may have been missed in those without testing. In addition, reliance on non-protocolised, clinically indicated investigations may have contributed to misclassification of myocardial injury, particularly in patients with isolated ECG abnormalities or incomplete cardiac assessment. These factors are central to interpreting the relatively high reported incidence of myocardial injury and support cautious interpretation of prevalence estimates. Accordingly, the reported incidence of myocardial injury should be interpreted cautiously.

Finally, the absence of long-term functional and cardiovascular follow-up limits assessment of outcomes beyond hospital discharge. In addition, multivariable analyses were exploratory in nature and may be subject to residual confounding or overfitting; no adjustment was made for multiple comparisons, and missing data were handled using complete-case analysis. Model performance was assessed using measures of discrimination (area under the receiver operating characteristic curve), without formal evaluation of calibration, which limits interpretation of predictive accuracy.

## Conclusions

In this single-centre cohort, myocardial injury was common following aneurysmal subarachnoid haemorrhage and was strongly associated with poorer neurological grade at presentation, higher rates of neurovascular complications, and increased ICU and hospital mortality; however, these associations likely reflect overall disease severity rather than a direct, modifiable causal effect of myocardial injury.

## References

[1] Messina A, Longhitano Y, Zanza C (2023). Cardiac dysfunction in patients affected by subarachnoid haemorrhage affects in-hospital mortality: a systematic review and metanalysis. Eur J Anaesthesiol.

[2] NA NA (2000). Epidemiology of aneurysmal subarachnoid hemorrhage in Australia and New Zealand: incidence and case fatality from the Australasian Cooperative Research on Subarachnoid Hemorrhage Study (ACROSS). Stroke.

[3] D'Souza S (2015). Aneurysmal subarachnoid hemorrhage. J Neurosurg Anesthesiol.

[4] Szántó D, Luterán P, Gál J, Nagy EV, Fülesdi B, Molnár C (2023). Diagnosis and management of Takotsubo syndrome in acute aneurysmal subarachnoid hemorrhage: a comprehensive review. Rev Cardiovasc Med.

[5] Osteraas ND, Lee VH (2017). Neurocardiology. Handb Clin Neurol.

[6] van der Bilt I, Hasan D, van den Brink R (2014). Cardiac dysfunction after aneurysmal subarachnoid hemorrhage: relationship with outcome. Neurology.

[7] Anetsberger A, Jungwirth B, Blobner M (2021). Association of troponin T levels and functional outcome 3 months after subarachnoid hemorrhage. Sci Rep.

[8] Mogollon JP, Smoll NR, Panwar R (2018). Association between neurological outcomes related to aneurysmal subarachnoid hemorrhage and onsite access to neurointerventional radiology. World Neurosurg.

[9] Sano H, Inamasu J, Kato Y, Satoh A, Murayama Y (2016). Modified world federation of neurosurgical societies subarachnoid hemorrhage grading system. Surg Neurol Int.

[10] Fisher CM, Kistler JP, Davis JM (1980). Relation of cerebral vasospasm to subarachnoid hemorrhage visualized by computerized tomographic scanning. Neurosurgery.

[11] Murthy SB, Shah S, Rao CP, Bershad EM, Suarez JI (2015). Neurogenic stunned myocardium following acute subarachnoid hemorrhage: pathophysiology and practical considerations. J Intensive Care Med.

[12] Wang J, Lin F, Zeng M (2024). Intraoperative blood pressure and cardiac complications after aneurysmal subarachnoid hemorrhage: a retrospective cohort study. Int J Surg.

[13] Rehman S, Chandra RV, Zhou K (2020). Sex differences in aneurysmal subarachnoid haemorrhage (aSAH): aneurysm characteristics, neurological complications, and outcome. Acta Neurochir (Wien).

[14] Yamada S, Ishikawa M, Yamamoto K, Ino T, Kimura T, Kobayashi S (2015). Aneurysm location and clipping versus coiling for development of secondary normal-pressure hydrocephalus after aneurysmal subarachnoid hemorrhage: Japanese Stroke DataBank. J Neurosurg.

[15] Sung SB, Kim YD, Ban SP, Lee YJ, Kwon OK (2022). Initial severity of aneurysmal subarachnoid hemorrhage (SAH): trend over time. J Cerebrovasc Endovasc Neurosurg.

[16] Goertz L, Kabbasch C, Styczen H (2021). Impact of aneurysm morphology on aneurysmal subarachnoid hemorrhage severity, cerebral infarction and functional outcome. J Clin Neurosci.

[17] Parekh N, Venkatesh B, Cross D (2000). Cardiac troponin I predicts myocardial dysfunction in aneurysmal subarachnoid hemorrhage. J Am Coll Cardiol.

[18] Yousef KM, Crago E, Lagattuta TF, Hravnak M (2018). Clinical presentation to the emergency department predicts subarachnoid hemorrhage-associated myocardial injury. J Emerg Nurs.

[19] de Courson H, Chadefaux G, Loiseau A, Georges D, Biais M (2023). Myocardial dysfunction assessed by speckle-tracking in good-grade subarachnoid hemorrhage patients (WFNS 1-2): a prospective observational study. Crit Care.

[20] Jeon IC, Chang CH, Choi BY (2009). Cardiac troponin I elevation in patients with aneurysmal subarachnoid hemorrhage. J Korean Neurosurg Soc.

[21] Mayer SA, Lin J, Homma S (1999). Myocardial injury and left ventricular performance after subarachnoid hemorrhage. Stroke.

[22] Urbaniak K, Merchant AI, Amin-Hanjani S (2007). Cardiac complications after aneurysmal subarachnoid hemorrhage. Surg Neurol.

[23] Zhao J, Gu S, Zhao X, Wang S, Pan Q, Zou C (2025). Prognostic value of elevated cardiac troponin in aneurysmal subarachnoid hemorrhage: a systematic review and meta-analysis. Front Neurol.

[24] Ahmadian A, Mizzi A, Banasiak M (2013). Cardiac manifestations of subarachnoid hemorrhage. Heart Lung Vessel.

[25] van der Bilt IA, Hasan D, Vandertop WP, Wilde AA, Algra A, Visser FC, Rinkel GJ (2009). Impact of cardiac complications on outcome after aneurysmal subarachnoid hemorrhage: a meta-analysis. Neurology.

[26] Naidech AM, Kreiter KT, Janjua N (2005). Cardiac troponin elevation, cardiovascular morbidity, and outcome after subarachnoid hemorrhage. Circulation.

[27] Zhang L, Zhang B, Qi S (2020). Impact of echocardiographic wall motion abnormality and cardiac biomarker elevation on outcome after subarachnoid hemorrhage: a meta-analysis. Neurosurg Rev.

[28] Oras J, Grivans C, Bartley A, Rydenhag B, Ricksten SE, Seeman-Lodding H (2016). Elevated high-sensitive troponin T on admission is an indicator of poor long-term outcome in patients with subarachnoid haemorrhage: a prospective observational study. Crit Care.

[29] Schuiling WJ, Algra A, de Weerd AW, Leemans P, Rinkel GJ (2006). ECG abnormalities in predicting secondary cerebral ischemia after subarachnoid haemorrhage. Acta Neurochir (Wien).

[30] Odensass S, Gümüs M, Said M (2024). Predictors of survival after aneurysmal subarachnoid hemorrhage: the long-term observational cohort study. Clin Neurol Neurosurg.

[31] Nieuwkamp DJ, Setz LE, Algra A (2009). Changes in case fatality of aneurysmal subarachnoid haemorrhage over time, according to age, sex, and region: a meta-analysis. Lancet Neurol.

[32] Mahlamäki K, Rautalin I, Korja M (2022). Case fatality rates of subarachnoid hemorrhage are decreasing with substantial between-country variation: a systematic review of population-based studies between 1980 and 2020. Neuroepidemiology.

[33] Ketelauri P, Gümüs M, Karadachi HH (2025). The impact of home medications on the risk of delayed cerebral ischemia after aneurysmal subarachnoid hemorrhage. Acta Neurochir (Wien).

[34] Djilvesi D, Nikolic D, Nikolic MB, Jelaca B, Golubovic J, Pajicic F, Lasica N (2025). Predictive model and scoring system for delayed cerebral ischemia following aneurysmal subarachnoid hemorrhage: a ten-year prospective analysis of observational data. Brain Spine.

[35] Malik AN, Gross BA, Rosalind Lai PM, Moses ZB, Du R (2015). Neurogenic stress cardiomyopathy after aneurysmal subarachnoid hemorrhage. World Neurosurg.

